# Staging of esophageal cancer using PET/MRI: a systematic review with head-to-head comparison

**DOI:** 10.1186/s12880-025-01565-9

**Published:** 2025-01-30

**Authors:** Alisa Mohebbi, Saeed Mohammadzadeh, Zahra Moradi, Afshin Mohammadi, Hossein Poustchi, Seyed Mohammad Tavangar

**Affiliations:** 1 Universal Scientific Education and Research Network (USERN), Tehran, Iran; 2 School of Medicine, Tehran University of Medical Sciences, Tehran, Iran; 3 Department of Radiology, Faculty of Medicine, Urmia University of Medical Science, Urmia, Iran; 4 Digestive Disease Research Institute, Tehran University of Medical Sciences, Tehran, Iran; 5 Department of Pathology, Dr. Shariati Hospital, Tehran University of Medical Sciences, Tehran, Iran; 6 Chronic Diseases Research Center, Endocrinology and Metabolism Population Sciences Institute, Tehran University of Medical Sciences, Tehran, Iran

**Keywords:** Positron emission tomography (PET), Magnetic resonance imaging (MRI), PET/MRI, PET/MR, Esophageal cancer

## Abstract

**Purpose:**

To evaluate the staging performance of positron emission tomography/magnetic resonance imaging (PET/MRI) for confirmed esophageal cancer based on the TNM classification system as well as compare it to other alternative modalities (e.g., endoscopic ultrasonography (EUS), computed tomography (CT), MRI, and PET/CT) in a full head-to-head manner.

**Methods:**

Protocol was pre-registered a priori at (http://osf.io/6qj5m/). We searched PubMed, Web of Science, Embase, and Cochrane Library for studies until September 10, 2024. The risk of bias was assessed using Modified Quality Assessment of Diagnostic Accuracy Studies (QUADAS-2) and Quality Assessment of Diagnostic Accuracy Studies–Comparative (QUADAS-C). The classification performance of PET/MRI in T, N, and M staging of esophageal cancer and resectability status were evaluated and compared to other relative modalities. Grading of Recommendations, Assessment, Development, and Evaluations (GRADE) was used for certainty evaluation.

**Results:**

Nine studies were included with 245 esophageal cancer patients. For T, N, and M staging, PET/MRI showed 9.1%, 2.0%, and 10.7% upstaging than the histopathological evaluation while these numbers were 19.4%, 12.4%, and 5.3% for downstaging. For direct comparison with PET/CT, PET/MRI showed 0.7% and 5.6% less downstaging and upstaging for N staging and 2.5% and 4.0% for M staging. As for predicting resectability status, pre-ADCmean and post-ADCmean were promising, unlike other parameters (i.e., ΔADCmean, pre-SUVmax, post-SUVmax, and ΔSUVmax).

**Conclusion:**

With protocol adjustments, PET/MRI might be utilized in the future for preoperative staging of esophageal cancer.

**Clinical trial number:**

N/A.

**Supplementary Information:**

The online version contains supplementary material available at 10.1186/s12880-025-01565-9.

## Introduction

Esophageal cancer ranks sixth among the most prevalent causes of cancer-related mortality worldwide [1]. The prognosis is generally unfavorable, with a 5-year survival rate of 15–25% due to late presentation [2]. The primary treatment for esophageal cancer invovles a combination of surgical intervention and neoadjuvant chemotherapy or chemoradiotherapy. Imaging modalities contribute substantially to the determination of accurate tumor staging, with computed tomography (CT), endoscopic ultrasonography (EUS), magnetic resonance imaging (MRI), and positron emission tomography (PET)/CT being commonly employed for this purpose [3]. The capability to predict the response to neoadjuvant therapy (NT) is of considerable value, as an inadequate response following NT may indicate therapy resistance, which can result in disease progression, unnecessary surgical delays, as well as the possibility that the tumor may become unresectable [4].

Recently developed hybrid PET/MRI is a rapidly evolving diagnostic modality whose full potential has yet to be discovered in the literature, and whose full diagnostic utility has yet to be proven. PET/MRI is used in the field of oncology to detect and stage tumors, evaluate treatment responses, and assess tumor recurrence [5]. This technique synergistically merges the functional information provided by PET with the high-resolution anatomical imaging features of MRI. Additionally, PET/MRI does not expose patients to ionizing radiation, further improving its safety aspects [6]. PET/MRI can detect superior soft tissue contrast, which can provide crucial information on tumor depth and nodal involvement, which is impossible with CT and PET/CT [6]. The uptake of the18F-Fludeoxyglucose (18 F-FDG) as a PET tracer can be precisely measured using the standardized uptake value (SUV), allowing the evaluation of metabolic activity within the tumor and the possibility of metastatic spread [7].

To our knowledge, no previous diagnostic systematic review has examined the role of PET/MRI for staging and determining the post-NT resectability status of esophageal cancer. This systematic review aims to assess the staging performance of PET/MRI and compare it with other imaging modalities.

## Materials and methods

The priori protocol registration of this study has been made at (http://osf.io/6qj5m/) (Appendix A) on the Open Science Framework (OSF) platform. The methodology was based on the Cochrane Handbook for Systematic Reviews of Diagnostic Test Accuracy [8]. We structured this study according to Preferred Reporting Items for a Systematic Review and Meta-analysis of Diagnostic Test Accuracy (PRISMA-DTA) and Search (PRISMA-S) (Appendix B).

### Search strategy

Two experienced reviewers, each having conducted at least ten meta-analyses, independently designed the search strategy. The two strategies were discussed and subsequently merged for each database. Disagreements were resolved by a third reviewer. To ensure comprehensive coverage, the search strategy incorporated both EMTREE and MeSH keywords and was applied to four databases: PubMed, Web of Science, Embase, and Cochrane Library. The search was conducted until September 10, 2024, without language restrictions (Appendix C). Furthermore, a review of the reference lists of relevant papers was also conducted to identify any missing publications. All retrieved records were imported into EndNote software version 18, and duplicate records were eliminated manually after automated checking.

### Eligibility criteria & study selection

We included case-control, cohort, cross-sectional, and clinical trial studies. The studies included in this review met the following criteria: (1) research involving more than five patients with histopathological confirmed esophageal cancer; (2) studies that employed hybrid (i.e., simultaneous) PET/MRI imaging. The exclusion criteria were as follows: studies utilizing sequential PET/MRI, studies lacking confirmation of primary esophageal cancer, non-observational studies (including case reports/series, editorials, comments, correspondence, and guidelines, as well as meta-analyses, systematic reviews, and narrative reviews), grey literature not published in peer-reviewed journals, and irrelevant publications. The main reason for excluding the studies utilizing the sequential PET/MRI is associated with potential misregistration errors and artifacts since sequential PET/MRI involves acquiring PET and MRI images separately, either on different machines or on the same machine at different times and can affect image interpretation and diagnostic accuracy. A study with more comprehensive data was chosen for evaluation when multiple publications utilized the same dataset. Study datasets that were subsets of another study’s dataset were considered less comprehensive. We cross-checked author names and countries of included studies to avoid including duplicate reports. Furthermore, the search results were independently screened by two reviewers, excluding irrelevant studies. Discrepancies between the reviewers were resolved through discussions. In the discussions that did not result in an agreement, a third reviewer also participated.

### Risk of bias assessment

Using the Modified Quality Assessment of Diagnostic Accuracy Studies (QUADAS-2) [9] and Quality Assessment of Diagnostic Accuracy Studies–Comparative (QUADAS-C) [10], two reviewers independently assessed bias risk in four domains of patient selection, index test, reference test, and flow and timing items. If the evaluators disagreed, they referred to a third reviewer.

### Data extraction

Data extraction was performed independently by two reviewers. The data was entered into an Excel sheet, and the files were cross-checked for consistency. For unresolved disagreements, the third reviewer was consulted. To evaluate PET/MRI performance for TNM staging, we compared each component of T, N, and M to the histopathological evaluation in three statuses of upstaging, downstaging, and same staging. When T, N, or M stages were detected at a higher stage in imaging than histopathology, it was considered upstaging, while downstaging was referred as the opposite. The T component was defined as T1, T2, T3, and T4 levels. The inter-stage assessment was defined to determine the down or upstaging. Meanwhile, intra-stage evaluation was used to evaluate the value of imaging modalities within a specific T stage. The N component was defined as N0 vs. N + levels. Patients with primary esophageal cancer without involvement of regional lymph nodes were considered N0, and those with involvement of one or more regional lymph nodes are considered N+. As for M component patients were categorized as having distant metastasis (M1) or without it (M0). To reach a robust consensus, PET/MRI was compared to other currently employed imaging modalities in a double-arm comparison (i.e., all included patients received multiple imaging modalities). Also, summary data for surgically resectable vs. unresectable patients for PET/MRI parameters such as apparent diffusion coefficient (ADC) and standardized uptake value (SUV) for both pre- and post-NT were extracted.

### Data synthesis

Statistical analyses were conducted using STATA version 17.0, MedCalc version 20.0. A random-effects model was employed. If included studies reported data for multiple groups separately, which are considered a single group based on our research question, we pooled the data for these groups separately and then pooled the outcome effect size with the effect size of other studies to avoid impact bias by that study [11]. The certainty of evidence was assessed using the Grading of Recommendations, Assessment, Development, and Evaluations (GRADE) tool. High statistical heterogeneity was considered as an I2 value of ≥ 50% [12]. Statistical significance was determined by a p-value of < 0.05.

## Results

### Search results and study characteristics

**Fig. 1:**
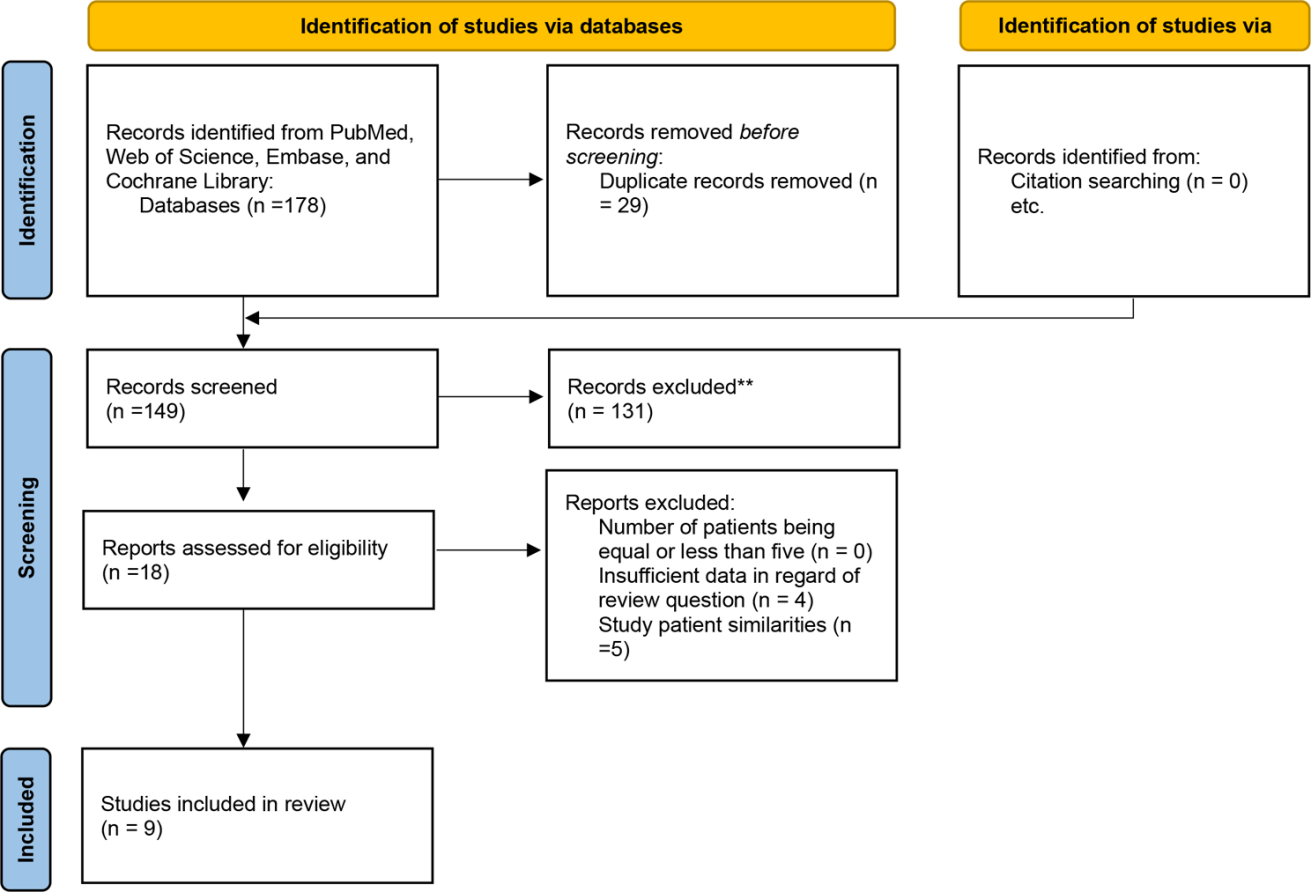
PRISMA flowchart. The leading causes for record exclusion** were: (**a**) studies that evaluated the prognostic value of PET/MRI in esophageal cancer (**b**) poor-quality conference abstracts (**c**) studies that performed sequential PET and MRI examinations

A total of nine studies were included with 245 esophageal cancer patients (Table 1). Of these, 5 studies (41% of patients (101/245)) were from Europe, and 4 studies (59% of patients (144/245)) were from East Asia. All studies were prospective in their methodological design. Also, no additional conference abstract studies were found. PRISMA flowchart is also shown in Fig. 1 [6, 7, 13–19].

**Table 1:** Characteristics of the included studies

First author (year)	Study type	Country or Region	Number of patients	Field strength	FDG dose	FDG acquisition start time	Number of radiologists with years of experience
Lee et al. (2014)	Prospective	Korea	15	3	3.7 MBq/kg	60 min	4
Belmouhand et al. (2018)	Prospective	Denmark	22	not reported	4 MBq/kg	60 min	2 with 12 and 15 YOE
Baiocco et al. (2019)	Prospective	United Kingdom- Italy	20	not reported	326 ± 28 MBq	156 ± 23 min	1 with > 5 YOE
Linder et al. (2019)	Prospective	Sweden	16	3	334 ± 48 mBq	109 ± 22 min	2 groups of radiologists
Yu et al. (2019)	Prospective	Taiwan	54	3	5 MBq/kg (0.14 mCi/kg)	105 min	2
Sharkey et al. (2021)	Prospective	United Kingdom	22	not reported	324 ± 28 MBq	112.5 ± 18.1 min	2
Wang et al. (2022)	Prospective	China	35	3	3.7 MBq/kg	60 ± 10 plus 30–40 min	2 experienced physicians
Chao et al. (2023)	Prospective	Taiwan	40	3	370MBq	114 min	2
Valkema et al. (2023)	Prospective	Netherlands	21	not reported	2.7 MBq/kg	93 min	4

### Risk of bias assessment

**Fig. 2:**
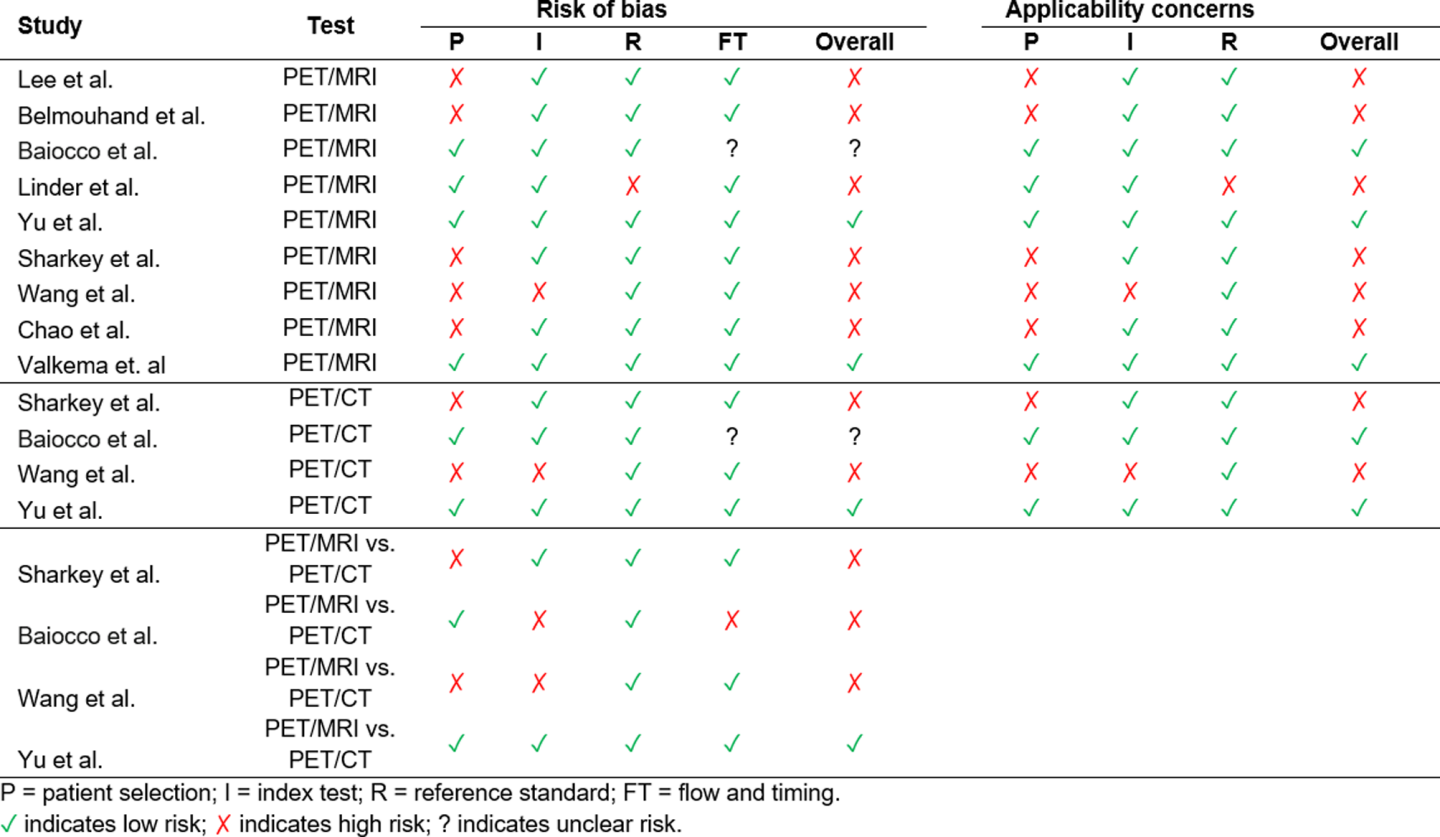
Risk of bias assessment based on Quality Assessment of Diagnostic Accuracy Studies-2 (QUADAS-2) and QUADAS-C

Figure 2 provides a visual representation of the risk of bias assessment based on QUADAS-2 criteria. As for the domains, both reference standard biases and index tests exhibited low bias patterns in the included studies. PET/MRI assessment and gold standard test were separated by a short time interval, so flow and timing bias was not substantial. The only reason for the selection bias observed in the included studies was the exclusion of patients with unresectable tumors. This may lead to downgrading bias, making their results trivial.

### T staging

**Fig. 3:**
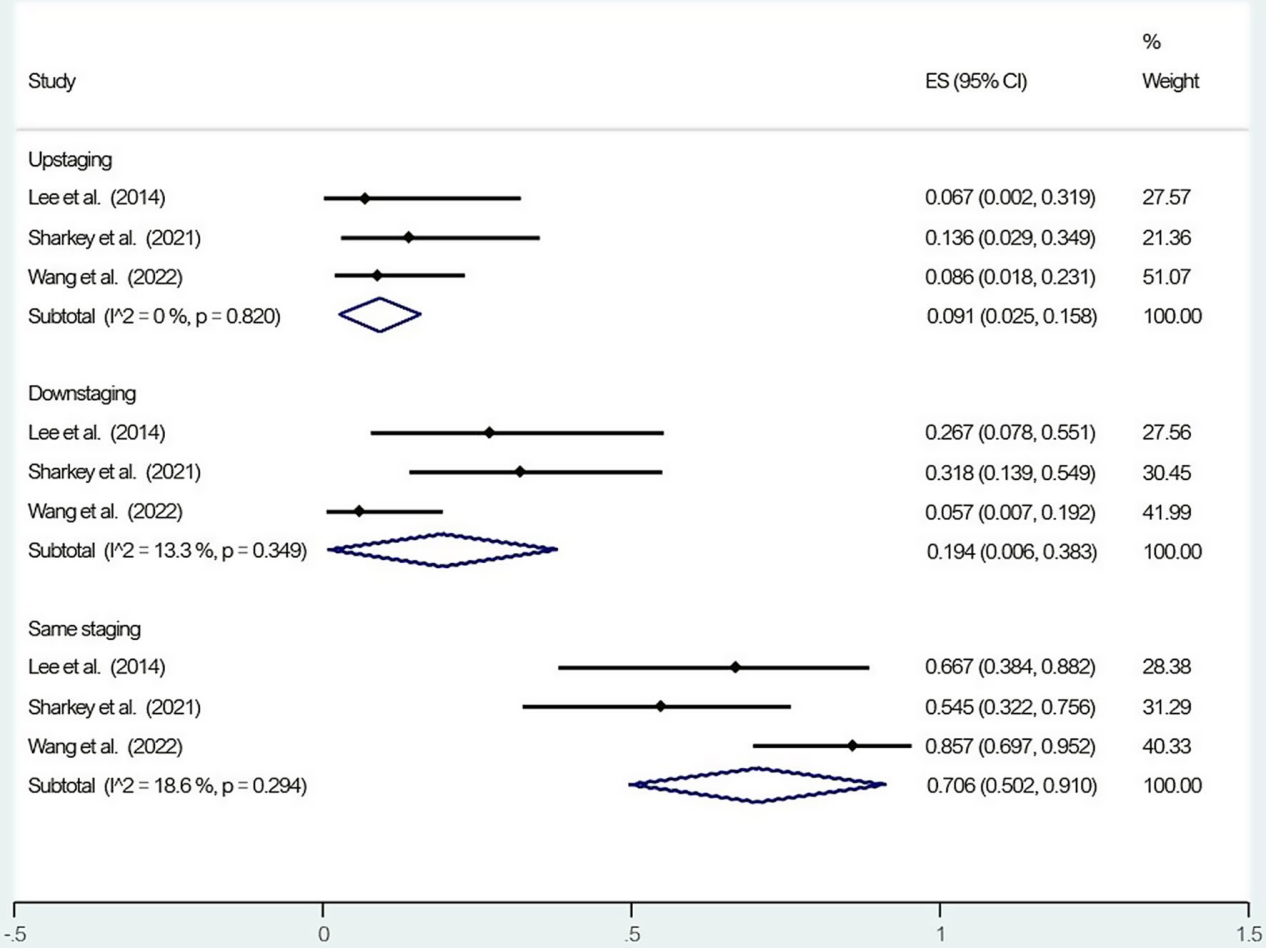
Forest plot of T inter-staging for positron emission tomography/magnetic resonance imaging (PET/MRI)

Figure 3 represents the pooled PET/MRI T inter-staging data. Compared to histopathological evaluation, PET/MRI showed 9.1% (95% confidence interval (CI) = 2.5–15.8%, I2 = 0%) upstaging, 19.4% (CI = 0.6–38.3%, I2 = 13.3%) downstaging, and 70.6% (CI = 50.2–91.0%, I2 = 18.6%) same staging. Regarding T intra-staging, PET/MRI accurately detected 84.1% (CI = 69.6–98.7%) of T1, 74.1% (CI = 48.8–99.4%) of T2, and 86.5% (CI = 70.5–100%) of T3 tumors compared to histopathological evaluation.

### N staging

**Fig. 4:**
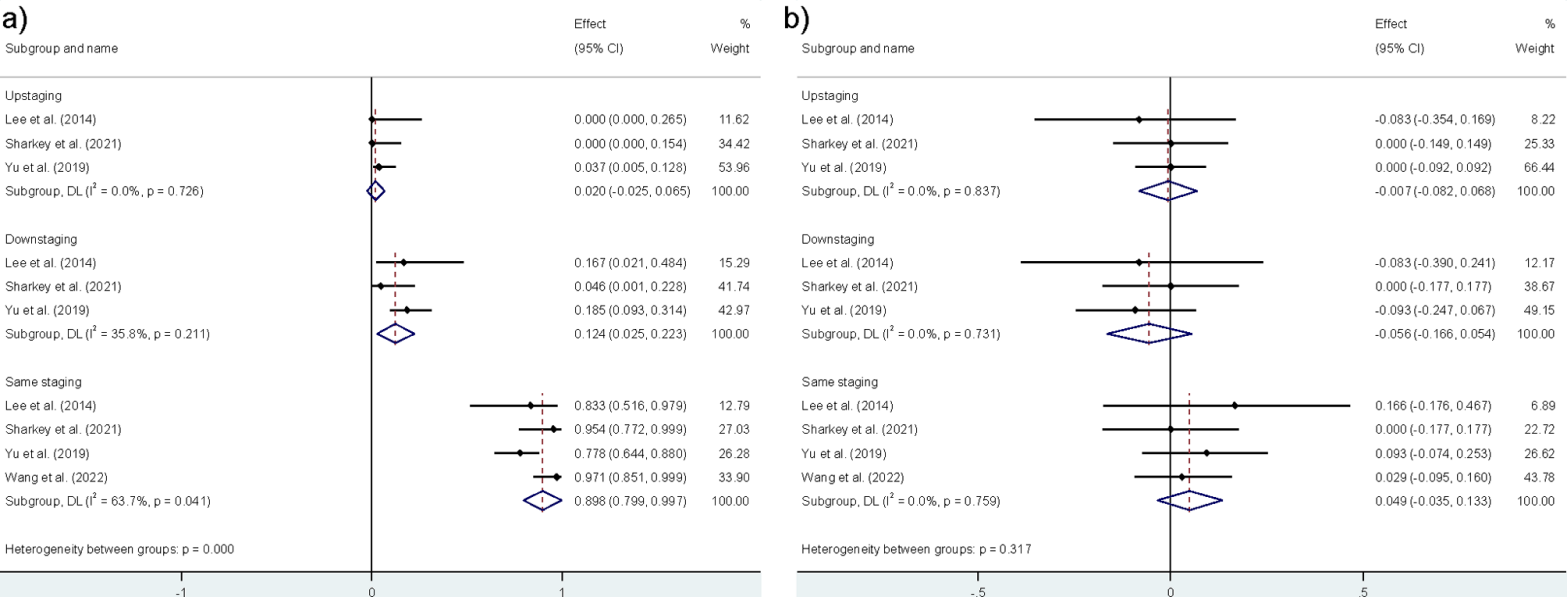
**A**) Pooled PET/MRI N staging data. **B**) Direct comparison of PET/MRI vs. positron emission tomography/computed tomography (PET/CT) in N staging

Figure 4A represents the pooled PET/MRI N staging data. Compared to histopathological evaluation, PET/MRI demonstrated 2.0% (CI = 0–6.5%, I2 = 0%) upstaging, 12.4% (CI = 2.5–22.3%, I2 = 35.8%) downstaging, and 89.8% (CI = 79.9–99.7%, I2 = 63.7%) same staging.

Figure 4B presents the comparison of PET/MRI with PET/CT in N staging. PET/MRI showed a lower upstaging and downstaging than PET/CT, with difference values of 0.7% (I2 = 0%) and 5.6% (I2 = 0%), respectively. Also, PET/MRI illustrated higher same staging compared to PET/CT with a difference value of 4.9% (I2 = 0%). These findings suggest that PET/MRI more closely aligns with histopathology for N staging.

### M staging

**Fig. 5:**
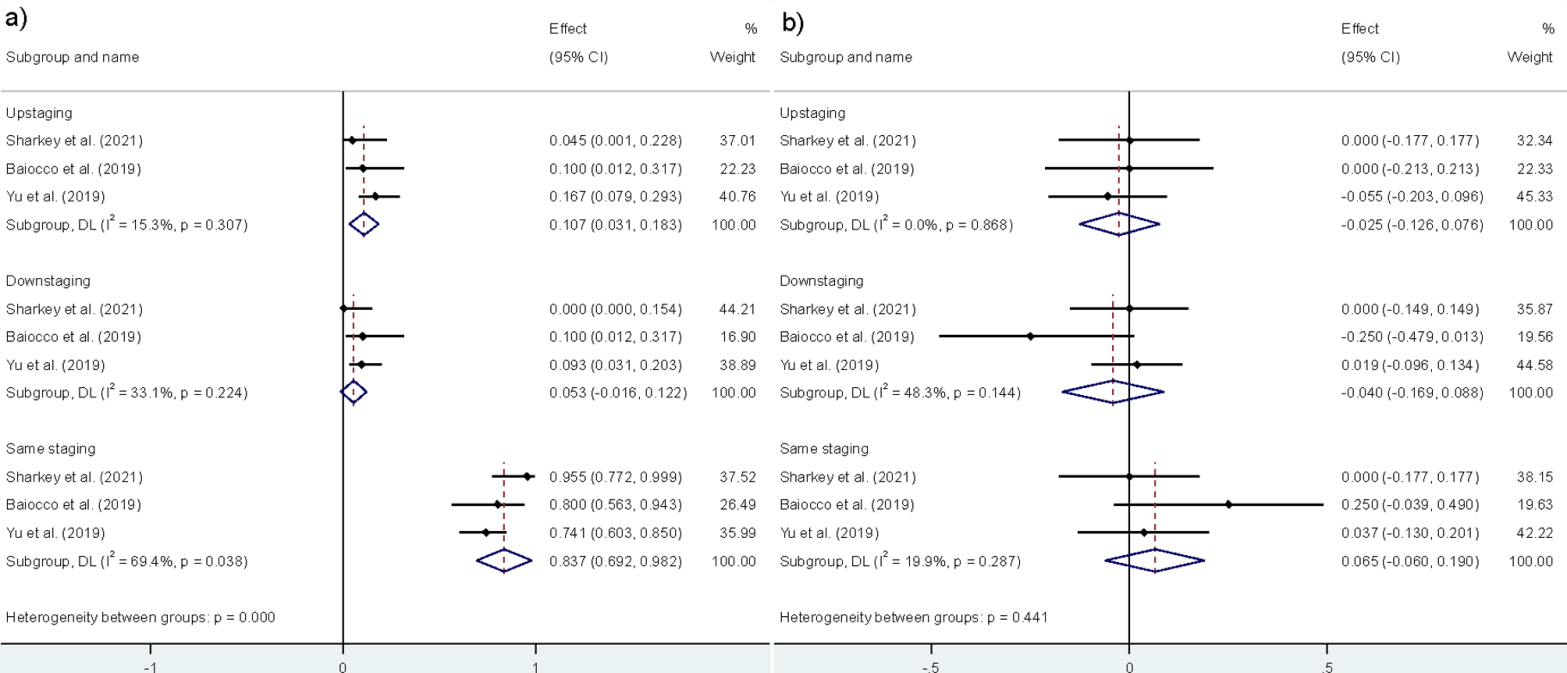
**A**) Pooled PET/MRI M staging data. **B**) Direct comparison of PET/MRI vs. PET/CT in M staging

Figure 5A represents the pooled PET/MRI M staging data. Compared to histopathological evaluation, PET/MRI demonstrated 10.7% (CI = 3.1–18.3%, I2 = 15.3%) upstaging, 5.3% (CI = 0–12.2%, I2 = 33.1%) downstaging, and 88.7% (CI = 69.2–98.2%, I2 = 69.4%) same staging.

Figure 5B depicts the comparison of PET/MRI vs. PET/CT in M staging. Similar to N staging, PET/MRI showed a lower upstaging and downstaging than PET/CT with difference values of 2.5% (I2 = 48.3%) and 4.0% (I2 = 19.9%), respectively. Furthermore, PET/MRI showed more same staging than PET/CT by a difference value of 6.5% (I2 = 19.9%). Comparing PET/MRI with PET/CT, these findings indicate that PET/MRI aligns more closely with histopathology for M staging. The summarized findings about the staging performance of PET/MRI for T, N, M staging can be found in Table 2.

**Table 2:** Staging performance of PET/MRI in T, N, M staging

Staging	Upstaging	Downstaging	Same staging
T staging	9.1% (CI = 2.5–15.8%)	19.4% (CI = 0.6–38.3%)	70.6% (CI = 50.2–91.0%)
N staging	2.0% (CI = 0–6.5%)	12.4% (CI = 2.5–22.3%)	89.8% (CI = 79.9–99.7%)
M staging	10.7% (CI = 3.1–18.3%)	5.3% (CI = 0–12.2%)	88.7% (CI = 69.2–98.2%)

### Systematic review on resectability

A total of four studies were included to evaluate the role of PET/MRI in determining resectability status [4, 10, 14, 15]. According to the literature review, PET/MRI imaging parameters of pre-ADCmean and post-ADCmean showed a promising value in determining the resectability status of tumors. Across all four studies, unresectable tumors exhibited lower pre- and post-ADCmean values compared to resectable tumors, suggesting that ADC could serve as a separating tool for resectable and unresectable tumors. However, other quantitative parameters (ΔADCmean, pre-SUVmax, post-SUVmax, and ΔSUVmax) showed controversial or trivial findings in determining resectability status.

As for 2 × 2 contingency table, only two studies reported it for PET/MRI parameters to determine eligibility for resection of primary esophageal cancer; Belmouhand et al. [6] reported a 93% sensitivity, 80% specificity, 81% accuracy and AUC of 0.95 for ΔSUVmax combined with ΔADCmean in determining resectability status of tumor while Sharkey et al. [16] reported 45% sensitivity, 80% specificity, 62% accuracy and AUC of 0.509 for the same parameters determining resectability status of tumor. Interpretation zones of AUCs were excellent in Belmouhand et al. [6] while unsatisfactory in Sharkey et al. [16] so more evaluation is needed to conclude in this regard.

### Certainty of evidence

On the basis of the GRADE method, Table 3 summarizes the certainty of evidence for the results of this study. N staging provided a higher level of certainty for the achieved results as compared to T and M staging for PET/MRI.

**Table 3:** Summary findings profile

Outcome	Number of studies	Number of patients	Diagnostic results	Risk of bias	Applicability and indirectness	Inconsistency	Imprecision	Publication bias	Strength of effect size	Certainty
PET/MRI performance for T staging	3	72	Figure 3	Moderate risk	Moderate risk	Consistent	Imprecise	Not applicable	Moderate Strength	Low ⊕⊕◯◯
PET/MRI performance for N staging	4	126	Figure 4	Moderate risk	Moderate risk	Consistent	Precise	Not applicable	High Strength	High ⊕⊕⊕⊕
PET/MRI performance for M staging	3	96	Figure 5	Moderate risk	Moderate risk	Consistent	Imprecise	Not applicable	High Strength	Moderate ⊕⊕⊕◯
PET/MRI performance for N staging compared to PET/CT	4	126	Figure 4	Moderate risk	Moderate risk	Consistent	Precise	Not applicable	High Strength	High ⊕⊕⊕⊕
PET/MRI performance for M staging compared to PET/CT	3	96	Figure 5	Moderate risk	Moderate risk	Consistent	Imprecise	Not applicable	High Strength	Moderate ⊕⊕⊕◯

## Discussion

**Fig. 6:**
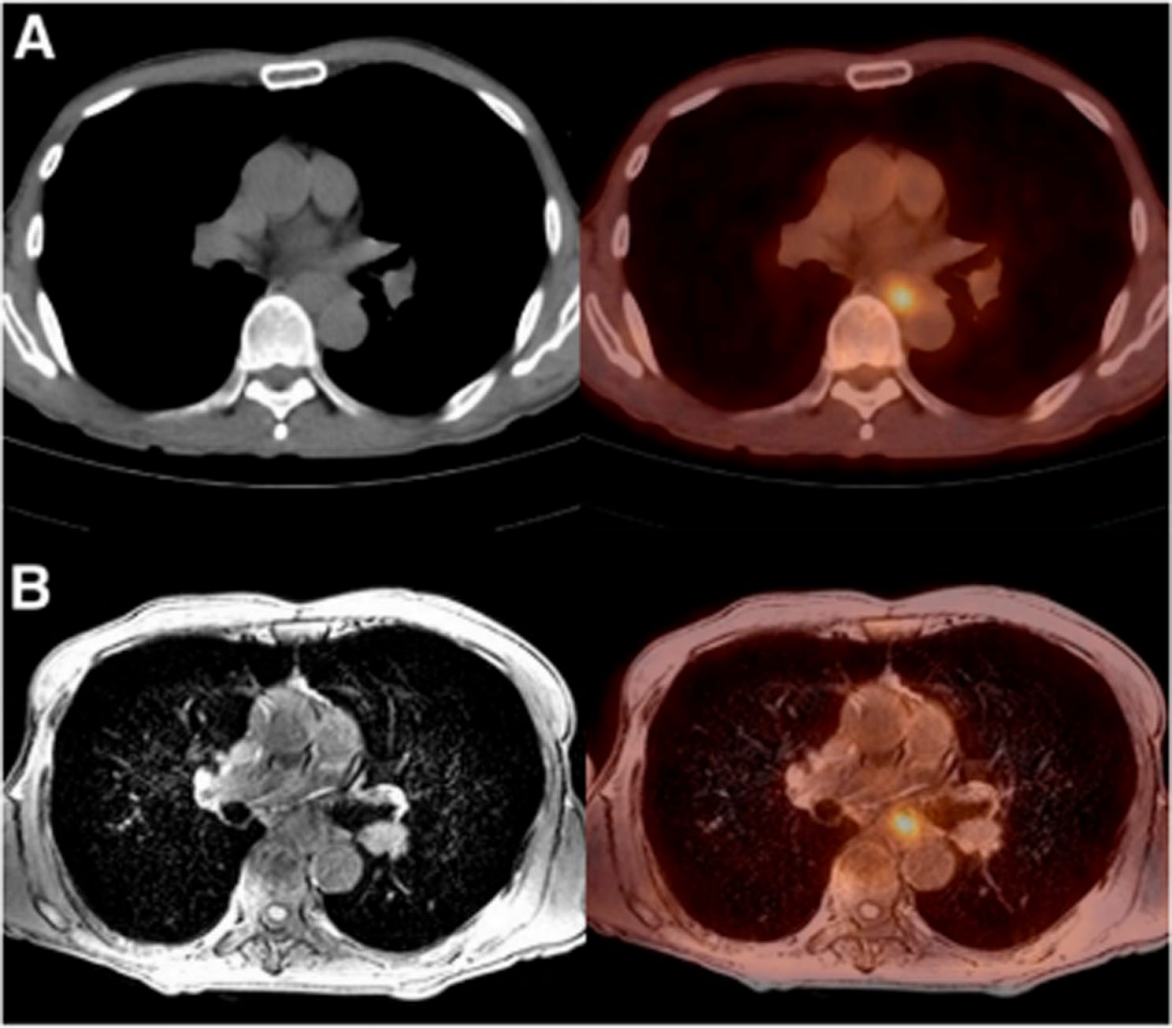
PET/CT (upper row) and PET/MRI (lower row) images of a representative case of esophageal cancer (a 63-year-old man). PET/MRI provides a higher quality image and better visibility than PET/CT [20]

Accurate determination of TNM stage is crucial, as down/upstaging of the tumor may lead to therapeutic resistance, disease advancement, unwarranted delay in surgery, overtreatment, increased risk of unresectability, etc [6]. Considering the poor prognosis of esophageal cancer and the new therapeutic options available, it is essential for physicians to accurately stage patients before surgery to make the best clinical decisions. Although CT, MRI, PET/CT, and EUS have limitations in assessing surgical resectability of tumors and detecting metastases from abdominal and thoracic lymph nodes, PET/MRI imaging offers distinct advantages. PET/MRI provides exceptional soft-tissue contrast that enables the visualization of esophageal wall stratification and the observation of surrounding tissue structure (20) (Fig. 6). Furthermore, PET/MRI allows for both qualitative assessment of anatomical structures and quantitative evaluation of both anatomical and metabolic activities associated with malignancies. Therefore, PET/MRI modalities have demonstrated significant advantages in achieving favorable outcomes in the differentiation of esophageal cancer, when compared to alternative approaches [21].

### T staging

Our findings indicate that PET/MRI achieved moderate diagnostic accuracy for T staging, with 70.6% same staging compared to the histopathological evaluation. The performance of PET/MRI in downstaging (19.4%) and upstaging (9.1%) was average. PET/MRI demonstrated superior results for N and M status perspectives compared to T staging. Also, in T intra-stage evaluation, PET/MRI diagnostic accuracy was slightly lower for the T2 stage compared to T1 and T3. However, none of the studies had reported patients with the T4 stage, so we could not assess the accuracy of PET/MRI at this stage.

In evaluating T intra-staging, four comparisons were made between EUS, CT, MRI, and PET/CT with PET/MRI. First, one study [13] directly compared PET/MRI diagnostic accuracy with EUS for each T intra-stage. The results demonstrated that for T1, T2, T3, PET/MRI had accuracy rates of 77%, 50%, 50%, while EUS exhibited higher accuracy rates of 88%, 50%, 100%.

Second, two studies assessed the diagnostic accuracy of PET/MRI compared to CT for T intra-stages. Lee et al. [13] reported accuracy rates of 77%, 50%, and 50% for T1, T2, and T3 in PET/MRI vs. 22%, 50%, and 50% for T1, T2, and T3 in CT. Similarly, Wang et al. [17] reported accuracy rates of 87%, 77%, and 90% for T1, T2, and T3 in PET/MRI vs. 40%, 44%, and 73% for T1, T2, and T3 in CT. These findings indicated that CT had poorer accuracy in T staging of tumors compared to PET/MRI, suggesting that PET/MRI may be a preferable alternative. Third, only one study [17] compared PET/MRI diagnostic accuracy with MRI for T intra-staging (87%, 77%, 90% for T1, T2, and T3 in PET/MRI vs. 66%, 77%, 90% for T1, T2 and T3 in MRI). MRI demonstrated poor diagnostic accuracy for T1 staging, leading to upstaging of most T1 tumors compared to PET/MRI. Fourth, since only one study [16] evaluated PET/CT diagnostic performance, we could not conduct a full head-to-head comparison as we did in N and M status for this imaging modality. Sharkey et al. [16] reported 0% upstaging and 63% downstaging for PET/CT. Comparing our PET/MRI pooled data to PET/CT, PET/CT performed poorly in downstaging. For a more robust conclusion, future studies need to examine the comparison in more detail.

For comparison of T inter-staging, only one study [13] compared PET/MRI with EUS (7% upstaging and 27% downstaging for PET/MRI vs. 7% upstaging and 7% downstaging for EUS). Although EUS showed superiority to PET/MRI in T staging, it is limited in many circumstances and cannot be used in patients with esophageal obstruction caused by tumors. PET/MRI could be used instead of EUS in these conditions. Only two studies compared PET/MRI with CT, making it impossible to perform meta-analysis as well. Lee et al. [13] reported 7% upstaging and 27% downstaging for PET/MRI vs. 27% upstaging and 40% downstaging for CT. Wang et al. [17] reported 9% upstaging and 6% downstaging for PET/MRI vs. 14% upstaging and 34% downstaging for CT. As for MRI, only one study [17] compared PET/MRI with it (9% upstaging and 6% downstaging for PET/MRI vs. 9% upstaging and 14% downstaging for MRI). Across these studies and a systematic review of them, PET/MRI has shown superior results in comparison to CT and MRI. However, studies comparing PET/MRI to other modalities especially, EUS and MRI were limited, and more evaluation is necessary to make robust conclusions.

### N staging

Based on our results, PET/MRI demonstrated high diagnostic performance for N staging, achieving an accuracy of 89.8%. PET/MRI upstaged N status more conservatively than downstaged it. Based on a fully head-to-head comparison of PET/MRI and PET/CT, the net benefit of using PET/MRI over PET/CT for 1000 confirmed esophageal cancer patients is a reduction in underdetection and overdetection of regional lymph node involvement by about 56 and 7 patients respectively (i.e., a considerable summed number of 63 patients are in favor of this technique). However, more studies are needed to obtain more robust statistical results as low numbers of studies cause inflation of this number, mainly due to the presence of a high level of risk of bias based on QUADAS-2 and QUADAS-C results mentioned above.

When comparing PET/MRI to other imaging modalities, only one study [13] compared PET/MRI with EUS, finding that PET/MRI had 0% upstaging and 16% downstaging, while EUS had 0% upstaging and 25% downstaging. Only two studies compared PET/MRI with CT, making it impossible to perform meta-analysis. Lee et al. [13] reported 0% upstaging and 16% downstaging for PET/MRI vs. 16% upstaging and 33% downstaging for CT. Wang et al. [17] reported 97% same staging accuracy for PET/MRI vs. 94% same staging accuracy for CT. Only two studies compared PET/MRI with MRI, making it impossible to perform meta-analysis. Yu et al. [15] reported 52% sensitivity and 100% specificity for PET/MRI vs. 94% sensitivity and 50% specificity for MRI. Wang et al. [17] reported 97% same staging accuracy for PET/MRI vs. 91% same staging accuracy for MRI. Based on the literature review, PET/MRI showed superior results compared to EUS, CT, and MRI; however, due to a limited number of studies comparing PET/MRI to other modalities, further study is needed before conclusions can be drawn.

### M staging

Our findings indicate that PET/MRI provides concise information in M staging with a strong 88.7% value for the same staging when compared to the histopathological evaluation. The downstaging and upstaging rates were 5.3% and 10.7% respectively. When applied to a cohort of 1000 confirmed esophageal cancer patients, the use of PET/MRI over PET/CT results in a reduction of metastasis downstaging and upstaging by about 40 and 25 patients (i.e., a considerable summed number of 65 patients are in favor of this technique).

In terms of the comparison of PET/MRI with other imaging modalities, only one study [14] directly compared PET/MRI with CT (45% upstaging and 5% downstaging for CT), and two studies compared PET/MRI with MRI, Baiocco et al. [14] reported 40% upstaging and 15% downstaging, while Yu et al. [15] reported 38.9% upstaging and no downstaging. Based on the findings of our study, PET/MRI demonstrated superior performance compared to CT and MRI in the field of upstaging and downstaging, but more research is needed to make a more reliable conclusion.

Also, only two studies [15, 17] evaluated the diagnostic performance of PET/MRI quantitative parameters (e.g., SUVmax, ADCmean) not only for resectability status but also in T, N, and M stagings. Previous articles suggested that pre- and post-ADC mean values can outperform traditional parameters such as SUVmax and might have a promising role as a separating tool for resectable and unresectable tumors. However, the studies evaluating quantitative parameters were sparse. Therefore, more studies are required to investigate and compare the diagnostic performance of these quantitative parameters.

Overall, our findings demonstrate that PET/MRI provides strong staging performance when compared to histopathological evaluations. This suggests that PET/MRI could play a pivotal role in clinical practice by assisting in cancer staging, predicting tumor resectability, and evaluating treatment response following chemotherapy.

### Limitations and future directions regarding PET/MRI

(1) This study included only nine articles, and the comparison of PET/MRI with other modalities such as EUS and PET/CT, as well as the evaluation of PET/MRI’s staging performance, was based on a limited number of studies. The limited number of articles raises concerns the robustness and generalizability of our conclusions. (2) The adoption of PET/MRI in clinical settings is currently hindered by restricted access to instrumentation and associated financial constraints. Limited availability of PET/MRI across healthcare institutions, coupled with the inherent cost of the technology, is a sound obstacle to widespread implementation [6]. (3) PET/MRI is limited by long acquisition imaging times (60–70 min) [15], MRI-incompatible metallic artifacts [7], and the substantial effect of even subtle breathing necessitate motion correction strategies [22]. (4) Currently, there is no widely accepted protocol for PET/MRI and only one study by Peerlings et al. [23] suggested a PET/MRI protocol for esophageal cancer. (5) As for prognostic goals, only one study [15] reported the hazard ratio for progression-free survival and overall survival of esophageal cancer.

Future studies should investigate the potential of other anatomical or functional parameters, such as total lesion glycolysis (TLG), metabolic tumor value (MTV), ADCmin, SUVpeak, K-trans, D, and pseudo-D. Both individual and combined effects of these parameters should be explored in prediction model studies. Furthermore, additional studies should investigate the impact of quantitative parameters, such as ADCmean and SUVmax, on cancer prognosis by assessing their predictive value for overall survival, progression-free survival, and recurrence rates. Validating these findings against histopathological results will further strengthen the evidence supporting the potential role of quantitative parameters from PET/MRI in assessing cancer staging and prognosis. Emphasis should be placed on high-quality, prospective, multicenter studies to minimize selection bias and ensure the reliability and generalizability of findings. Additionally, future research on diagnostic performance should involve direct comparisons with other modalities such as PET/CT or EUS.

## Conclusion

PET/MRI has demonstrated promising diagnostic performance in accurately staging esophageal cancer based on the TNM classification system. With adjustments in protocols, PET/MRI might play a role in preoperative esophageal cancer staging.

## Electronic supplementary material

Below is the link to the electronic supplementary material.

Supplementary Material 1

Supplementary Material 2

Supplementary Material 3

## Data Availability

Data generated in this study are available from the corresponding author upon request.

## Declarations

### Animal study

N/A.

## Abbreviations

ADC: Apparent diffusion coefficient
EUS: Endoscopic ultrasonography
18F: FDG-18 F-Fludeoxyglucose
NT: Neoadjuvant therapy
PET/CT: Positron emission tomography/computed tomography
PET/MRI: Positron emission tomography/magnetic resonance imaging
SUV: Standardized uptake value
