# Supplementary material for: Staging of esophageal cancer using PET/MRI: a systematic review with head-to-head comparison
Source: BMC Med Imaging. 2025 Jan 30;25:32. doi: 10.1186/s12880-025-01565-9 (PMC11783729; doi:10.1186/s12880-025-01565-9)
Supplement: Supplementary file 1 — Supplementary Material 1 [file 12880_2025_1565_MOESM1_ESM.pdf]

PET/MRI in determining esophageal cancer stage and resectability: A systematic review and meta-analysis.

1. Review question:

To determine the performance of PET/MRI in esophageal cancer staging and resectability

2. Searches:

Reviewers A.Mohebbi., S.M. and I.K. will develop their search syntax independently. In addition, EMTREE and MeSH keywords will be checked. They will merge their strategies after discussing the differences. If the discussion does not lead to a conclusion, a fourth reviewer (S.M.T) will be involved. A finalized search syntax will be conducted on December 20, 2023, and all eligible studies will be included. We will search PubMed, Web of Science, Embase, and Cochrane Library databases. Language restrictions will not be applied. Additionally, conference abstracts will be included. To ensure no publications are missed, the reference lists of relevant papers will also be reviewed. To perform screening, all searched studies will be imported into EndNote software. After that, duplicate studies will be removed.

((PET-MRI) OR (PET/MRI) OR (PET-MR) OR (PET/MR) OR (MRI-PET) OR (MRI/PET) OR (MR-PET) OR (MR/PET)) AND (esophagus OR esophageal OR oesophagus OR oesophageal OR gastroesophageal OR esophagogastric)

3. Types of study to be included:

Case-control, cohort, cross-sectional, and clinical trials with more than five patients will be included.

4. Condition or domain being studied:

Surgical intervention, with or without neoadjuvant chemotherapy or chemoradiotherapy (NT), remains to be the primary treatment for esophageal cancer. The process of patient selection and prognostic prediction in individuals diagnosed with esophageal cancer is critical for decisions on patient treatment. The ability to predict the response to neoadjuvant therapy holds significant value, as an inadequate response following NT may indicate therapy resistance, which can result in disease progression, unwarranted surgical delays, and the potential for unresectable tumors. Effective tumor staging heavily relies on the utilization of imaging techniques. The imaging modalities commonly employed for staging purposes encompass CT, endoscopic ultrasonography (EUS), and PET/CT.

Recently developed hybrid PET/MR imaging combines metabolic and anatomic information about cancer offering a comprehensive and integrated approach. PET/MR imaging is a rapidly

evolving diagnostic modality whose full potential has yet to be discovered in the literature, and full diagnostic utility has yet to be proven. In oncology, PET/MRI aids in the detection and staging of tumors, evaluation of treatment response, and assessment of tumor recurrence.for accurate disease diagnosis and monitoring. PET/MR imaging was shown to be capable of detecting superior soft tissue contrast, which can provide crucial information on tumor depth and nodal involvement , whereas such use was limited for CT and impossible for PET/CT. The uptake of the PET tracer (18F-FDG) can be precisely measured using the standardized uptake value (SUV), enabling the evaluation of metabolic activity within the tumor and the presence of metastatic manifestations.

5. Participants/population:

Patients who have confirmed esophageal cancer and have undergone PET/MRI will be examined.

6. Intervention(s), exposure(s):

This study will be conducted to determine the effectiveness of PET/MRI in the TNM staging system components and resectability of esophageal cancer.

7. Comparator(s)/control:

We performed this study to determine whether PET/MRI can effectively diagnose and stage esophageal cancer using the TNM staging system components.

8. Main outcome:

To form 2x2 contingency table for determining the resectability of esophageal cancer for included studies for each of the PET or MRI parameters and then calculate the diagnostic performance parameters (e.g., positive likelihood ratio, negative likelihood ratio, sensitivity, specificity, diagnostic odds ratio)

9. Additional outcome(s):

A) To perform contingency table for each three components of the TNM staging system for included studies for each of the PET or MRI parameters and then calculate the diagnostic performance parameters if enough data are reported.

B) To compare overall diagnostic performance of PET/MRI with current imaging modalities such as EUS, PET/CT and CT if enough data are reported.

C) To determine the best cutoff point for PET/MRI parameters if enough data are reported in studies.

D) To evaluate agreement parameters (e.g., kappa, ICC, CCC,) if enough data are reported.

E) To perform publication bias evaluation on upper results.

F) To perform sensitivity analysis on upper analyses.

G) To investigate the possible sources of statistical heterogeneity and perform subgrouping based on QUADAS-2.

H) To perform GRADE assessment on upper results

10. Data extraction (selection and coding):

In order to answer the review question, three reviewers (A.M. and S.M. and I.K.) will independently extract data from the included studies. In the next step, the data will be entered into an Excel spreadsheet, and the three files will be compared. If disagreements cannot be resolved between them, a fourth reviewer (S.M.T) will be involved.

11. Risk of bias assessment:

Quality Assessment of Diagnostic Accuracy Studies-2 (QUADAS-2) will be used to assess bias of included studies, along with some additional comments recommended by Cochrane Handbook for Systematic Reviews of Diagnostic Test Accuracy.

12. Strategy for data synthesis:

The meta-analysis will be conducted using STATA version 17.0. A random-effects model will be used, as recommended by the Cochrane Handbook for Systematic Reviews of Diagnostic Test Accuracy for diagnostic studies. The $I2 \geq 50\%$ will be considered a high statistical heterogeneity model.

12. Contact details for further information:

Seyed Mohammad Tavangar

tavangar@ams.ac.ir

13. Organizational affiliation of the review:

Universal Scientific Education and Research Network (USERN), Tehran, Iran.

Students’ Scientific Research Center (SSRC), Tehran, Iran.

14. Review team members and their organizational affiliations:

Alisa Mohebbi

Universal Scientific Education and Research Network (USERN), Tehran, Iran.

Students’ Scientific Research Center (SSRC), Tehran, Iran.

Saeed Mohammadzadeh

Universal Scientific Education and Research Network (USERN), Tehran, Iran.

Students’ Scientific Research Center (SSRC), Tehran, Iran.

Iman Kiani

Universal Scientific Education and Research Network (USERN), Tehran, Iran.

Students’ Scientific Research Center (SSRC), Tehran, Iran.

15. Type and method of review:

Diagnostic test accuracy (DTA) systematic review and meta-analysis

16. Anticipated or actual start date:

December 20, 2023

17. Anticipated completion date:

February 1, 2024

18. Funding sources/sponsors:

None

19. Conflicts of interests:

None
