# Supplementary material for: Staging of esophageal cancer using PET/MRI: a systematic review with head-to-head comparison
Source: BMC Med Imaging. 2025 Jan 30;25:32. doi: 10.1186/s12880-025-01565-9 (PMC11783729; doi:10.1186/s12880-025-01565-9)
Supplement: Supplementary file 3 — Supplementary Material 3 [file 12880_2025_1565_MOESM3_ESM.docx]

| Searched database | Search syntax |
| --- | --- |
| PubMed and Cochrane library | ((PET-MRI) OR (PET/MRI) OR (PET-MR) OR (PET/MR) OR (MRI-PET) OR (MRI/PET) OR (MR-PET) OR (MR/PET)) AND (esophagus OR esophageal OR oesophagus OR oesophageal OR gastroesophageal OR esophagogastric) |
| Web of Science and Embase | ((PET-MRI) OR (PET-MR) OR (MRI-PET) OR MR-PET)) AND (esophagus OR esophageal OR oesophagus OR oesophageal OR gastroesophageal OR esophagogastric) |

All databases were searched through Ovid simultaneously.
